# Tuberculosis-HIV treatment with rifampicin or rifabutin: are the outcomes different?

**DOI:** 10.1590/0074-02760180420

**Published:** 2019-02-11

**Authors:** Carolina Arana Stanis Schmaltz, Fernanda de Oliveira Demitto, Flavia Marinho Sant’Anna, Valeria Cavalcanti Rolla

**Affiliations:** Fundação Oswaldo Cruz-Fiocruz, Instituto Nacional de Infectologia Evandro Chagas, Laboratório de Pesquisa Clínica em Micobactérias, Rio de Janeiro, RJ, Brasil

**Keywords:** tuberculosis, HIV, rifampicin, rifabutin, rifamycin, outcomes

## Abstract

**BACKGROUND:**

Rifamycins are a group of antibiotics mainly used in the treatment of tuberculosis (TB), however they interact with antiretroviral therapy (ART). Rifabutin allows more regimens options for concomitant imunodeficiency virus (HIV) treatment compared to rifampicin.

**OBJECTIVE:**

Compare the outcomes of TB-HIV co-infected patients who used rifampicin or rifabutin.

**METHODS:**

We analysed data from a prospective cohort study at National Institute of Infectious Diseases Evandro Chagas, Rio de Janeiro (RJ), Brazil. Patients who were treated for TB and HIV with rifampicin or rifabutin, from February 2011 to September 2016 were included.

**FINDINGS:**

There were 130 TB-HIV patients, of whom 102 were treated with rifampicin and 28 with rifabutin. All patients in the rifabutin-treated group and 55% of the rifampicin-treated group patients were ART-experienced. Patients treated with rifampicin had similar abandon and cure rates, interruptions in treatment due to adverse reactions, immune reconstitution inflammatory syndrome and a similar mortality rate as those treated with rifabutin. However, rifampicin-treated patients had higher CD4 counts and more frequently undetectable HIV viral load by the end of treatment (67% versus 18%, p < 0.001) compared to rifabutin-treated patients, even when only ART-experienced patients were evaluated (66,6% versus 36,3%, p = 0.039).

**CONCLUSIONS:**

Patients who used rifabutin had worst immune and virological control. This group had more ART-experienced patients. New and simpler regimens are needed for patients who do not respond to previous antiretroviral therapies.

One of the challenges of tuberculosis (TB) treatment in individuals with both TB and imunodeficiency virus (TB-HIV) is the drug-drug interaction between rifampicin (RMP) and antiretroviral therapy (ART). RMP-based regimens can be used concomitantly with efavirenz or nevirapine (non-nucleoside reverse transcriptase inhibitors ― NNRTI), raltegravir (integrase inhibitor) or lopinavir-ritonavir (protease inhibitor ― PI).^1^ ^,^ ^2^ ^,^ ^3^ However, when treatment with other ritonavir-boosted PI regimen is necessary, sub-therapeutic levels of PIs occur due to the increased metabolism of hepatic cytochrome P450 (CYP) enzymes by RMP.^4^ ^,^ ^5^ Consequently, the treatment of TB-HIV patients who do not favourably respond to first-line ART is extremely challenging.

Rifamycins are the most important class of drugs used to treat TB, with RMP as the preferred first-line of therapy, followed by rifabutin (RFB).^5^ ^,^ ^6^ Loeliger et al.^7^ reviewed both randomised controlled trials and cohort studies of RFB for TB treatment and suggested that although limited, there is evidence that RFB is as safe and effective as RMP for the treatment of TB.

Due to the cost of RFB, in Brazil it is primarily used in situations where RMP is inappropriate. Since there are no fixed dose combination regimens with RFB available,^8^ ^,^ ^9^ when patients are on a rifabutin-regimen they have to be carefully evaluated, because adherence can be more difficult. Concomitant use of ritonavir-boosted PIs increases RFB concentration; therefore, a lower dose of RFB is prescribed^10^ and ART adherence should be monitored to avoid severely low RFB concentrations.^7^ TB treatment failure and relapse due to acquired rifamycin resistance are described, mainly when an intermittent RFB regimen is utilised.^11^ ^,^ ^12^

The best concomitant treatment for both TB-HIV remains unknown, particularly for ART-experienced patients, since all of the alternatives available have disadvantages. The aim of this study was to analyse the outcomes of HIV patients on ART, treated with RMP for TB for the entire duration of anti-TB therapy, or with RFB during at least part of the treatment period.

## SUBJECTS AND METHODS

We analysed data from an ongoing prospective cohort study, described elsewhere,^13^ carried out at the National Institute of Infectious Diseases Evandro Chagas (INI), previously called Instituto de Pesquisa Clínica Evandro Chagas (IPEC), Fiocruz, a reference hospital for infectious diseases in Rio de Janeiro, Brazil. Patients who were concomitantly treated for TB and HIV, from February 2011 to September 2016, and used RMP or RFB as part of their TB treatment were included after signing a written informed consent form. Patients with resistance to rifamycins were excluded. The Committee on Ethics in Research of INI approved the study.

A diagnosis of TB was made when *Mycobacterium tuberculosis* culture was positive in any sample collected or when there was a positive smear or when suggestive imaging analysis (x-ray or computed tomography) showed positive results and clinical signs and symptoms were present and improved with TB treatment. Patients included without a positive culture were considered with a positive therapeutic response if they have improved signs, symptoms and imaging analysis results after the intensive therapy phase.

During the baseline visit, TB therapy was prescribed after data collection, which included sex, age, marital status, monthly income, education, alcohol use (using CAGE questionnaire), drug use, history of smoking (current or past), sexual behaviour, weight loss, clinical presentation of TB, the date of first positive HIV serology, history of a previous episode of TB, current and previous ART used. In addition, results of laboratory tests such as microscopic examination of sputum, culture of sputum, blood and other clinical specimens for mycobacteria and fungal pathogens, blood cell counts, serum levels of creatinine, albumin and liver enzymes, CD4 cell count and HIV viral load were recorded. ART was also adjusted when necessary in previously experienced patients.

Data recorded in follow-up visits included: clinical manifestations associated with TB or HIV infection, the occurrence of immune reconstitution syndrome (IRIS), adverse reactions related to ART or TB therapy, subsequent laboratorial tests results including CD4 cell count and HIV viral load and resistance to TB drugs. ART was initiated in naïve patients during follow-up visits after at least 15 days of TB treatment. Patients were followed-up until the end of TB treatment. After the end of TB treatment, patients continued to be examined in an HIV outpatient clinic at INI-Fiocruz, and if a TB relapse was suspected, they were referred to our TB outpatient clinic.

ART was offered according to contemporary Brazilian National Guidelines.^8^ ^,^ ^9^ The first-line anti-TB regimen most used during the study period was the combination of RMP or RFB, isoniazid, pyrazinamide and ethambutol. TB treatment was changed in cases of severe adverse reactions and drug resistance. RMP, with a weight-adjusted dose, was used when a NNRTI, raltegravir or lopinavir-ritonavir (400-400 mg b.i.d. or 800-200 mg b.i.d.) were prescribed as part of ART. RFB at a dose of 150 mg daily was used when the standard dose of lopinavir-ritonavir or other ritonavir-boosted PI were prescribed. Patients who used RFB for any period during TB treatment were allocated into the RFB group. All TB drugs were taken daily and were self-administered.

Chi-square and Fisher’s exact test were used to compare the distribution of categorical variables among patients who received RMP for the entire duration of anti-TB therapy with those who received RFB during at least part of the treatment duration. The distribution of continuous variables was compared using Wilcoxon’s rank sum test or analysis of variance (ANOVA). Two-sided p ≤ 0.05 was considered statistically significant. Data was analysed with SPSS 17.0 programme.

## RESULTS

Among the 130 TB-HIV patients studied, 102 were treated with RMP and 28 with RFB. Among these 28 patients treated with RFB, 18 started on RMP and changed to RFB to accommodate their ART regimen or because of efavirenz resistance (11 after the intensive phase of TB treatment), two were started on RFB and changed to RMP after adjusting their lopinavir-ritonavir dosage, two were started without rifamycin and changed to a regimen with RFB, and six used RFB from the beginning until the end of the TB treatment period. Median duration of TB treatment was 191 days (IQR 158-254) and was significantly different between the RMP (189 days) and RFB treatment groups (219 days; p = 0.034). However, when analysing only the days that patients in the RFB group received this medication, the median duration of RFB treatment was 172 days (IQR 125-259).

Among the 130 patients included, 60 were ART-naïve and 70 were ART-experienced. The median start day of ART after TB treatment initiation in naïve patients was 28 days (IQR 14-33). ART-experienced patients were using ART for a median of five years (IQR 2-9). All of the 28 patients in the RFB group used a ritonavir-boosted PI regimen during TB treatment; however, three started with efavirenz and RMP and changed to a ritonavir-boosted PI regimen and RFB (two due to primary resistance to efavirenz and one due to an adverse reaction). Eighty-five patients used RMP with efavirenz, one with nevirapine, four with raltegravir and 12 with the double of standard dose of lopinavir-ritonavir.

Patients treated with RMP had more years of education, used to smoke more, had lower serum albumin levels, and more frequently presented with disseminated TB (Table). There were more ART-naïve patients and without a previous TB episode in the RMP treatment group (Table). However, the default rate of previous TB episodes was not significantly different between patients who used RMP (22,7%) or RFB (10%; p = 0.37). All other characteristics were similar between both groups (Table). Median initial CD4 cell count was 177 cells/mm^3^, and was not significantly different between the groups (Table).

Twenty-one patients (23%) with a positive culture for *M. tuberculosis* abandoned treatment, one died and 69 (76%) were cured. Six patients included without a positive culture abandoned treatment (15%) and one died. The other 32 (82%) patients without a positive culture improved clinical signs and symptoms during the intensive phase of TB therapy and were considered TB cases. All were considered cured at the end of the TB treatment. There were no significant differences in the number of cured patients (p = 0.29), deaths (p = 0.51) and default (p = 0.22) rates between patients with or without a positive culture.

Patients treated with RMP had a similar TB treatment default and cure rates, interruption of therapy due to adverse reactions and IRIS as those treated with RFB in our cohort (Table). In terms of ART response, patients had higher median CD4 cell counts, lower median HIV viral load and more frequently undetectable HIV viral load by the end of treatment in the RMP group (Table).

When we compared the RFB treated group to ART-experienced patients in the RMP group, we observed a better improvement in the immune and virological response in the RMP group. There were 57 ART-experienced patients in the RMP group and 24 had done HIV viral load in the end of TB treatment. Of these 24 patients, 16 (66,6%) had undetectable viral load. These 16 patients used NRTI + NNRTI (14 efavirenz and one nevirapine) or NRTI + raltegravir. Twenty-two patients (of 28) who used RFB had done HIV viral load in the end of TB treatment and only eight (36,3%) had undetectable viral load, significantly less compared to the RMP group (p = 0.039). All these 22 patients used NRTI + PI.

We found no difference in mortality between the groups. There were two TB related deaths, both from the RMP-treated group (Table).

**TABLE:** Distribution of baseline variables and outcomes between imunodeficiency virus-tuberculosis (HIV-TB) patients treated with highly active antiretroviral therapy (HAART) and rifampicin or rifabutin

	Rifampicin (%) n = 102	Rifabutin (%) n = 28	p-value^*a*^
White race	44 (43)	8 (29)	0.12^*b*^
Age, median (IQR)	36 (30-44)	36.5 (32-48)	0.18
Male sex	74 (72)	18 (64)	0.27
Married	34 (33)	6 (21)	0.16
Men who have sex with men	38 (37)	8 (29)	0.27
Alcohol use (129)	33 (32)	10 (37)	0.40
Current or previous smoke history (127)	60 (59)	10 (38)	0.04
Drug use (129)	33 (32)	7 (26)	0.35
School education > 9 years	58 (57)	11 (39)	0.07
Monthly income < U$500.00 (113)	72 (83)	23 (88)	0.36
Previous TB episode	22 (22)	12 (43)	0.02
TB clinical presentation			0.04
Pleural-pulmonary	55 (53)	22 (79)	
Extra-pulmonary	13 (13)	1 (4)	
Disseminated	34 (33)	5 (18)	
Weight loss > 10%	76 (74)	19 (68)	0.32
Positive smear	45 (44)	12 (43)	0.54
Positive culture for *Mycobacterium tuberculosis*	74 (72)	17 (61)	0.16
Hemoglobin > 9 g% (128)	75 (76)	24 (86)	0.17
Serum albumin > 3 g% (111)	35 (41)	16 (64)	0.03
Resistance to at least one TB drug used except rifamycin	3 (3)	2 (7)	0.29
Previous HIV diagnosis	63 (62)	26 (93)	0.001
HAART naïve	58 (57)	3 (11)	< 0.001
Initial CD4 cell count, median (IQR) (cels/mm3) (111)	177 (64-350)	154 (31-296)	0.16^b^
Initial viral load, median (IQR) (log) (110)	4.6 (2.3-5.3)	4.0 (2.6-5.2)	0.46^b^
Immunereconstitution syndrome (IRIS)	8 (8)	2 (7)	0.63
TB treatment interruption due to adverse reactions	7 (7)	2 (7)	0.62
TB treatment default	22 (22)	5 (18)	0.45
TB related death	2 (2)	0 (0)	0.61
TB cure	78 (76)	23 (82)	0.36
Final CD4 cell count, median (IQR) (cels/mm3) (90)	324 (181-538)	188 (124-300)	0.011^b^
Final viral load, median (IQR) (log) (90)	0 (0-1.86)	3.4 (1.8-4.76)	< 0.001^b^
Undetectable final viral load (90)	46 (67)	4 (18)	< 0.001
TB relapse	1 (1)	0 (0)	0.78

*a*: χ^2^ test, Fisher’s exact test unless specified; *b*: Wilcoxon’s rank sum test; IQR: interquartile range.

## DISCUSSION

In Brazil, RFB is used to treat patients on ART that do not qualify for RMP, mainly those using ritonavir-boosted PI regimens different from lopinavir-ritonavir regimens with doses adjusted to accommodate RMP use.^8^ ^,^ ^9^ Many of that patients who use RFB started treatment with RMP, in fixed-dose pills containing ethambutol, pyrazinamide and isoniazid. Perhaps the physician’s decision to wait until the end of the intensive treatment phase avoiding an early deconstruction of TB regimen into separate pills, to switch just to a regimen with two drugs (RFB and isoniazid), may have helped in the understanding of the drug prescription and adherence. This strategy, together with the fact that lopinavir-ritonavir are prescribed in adjusted doses to allow for RMP use in fixed-dose combinations,^14^ possibly explains the similar default rate observed in both groups in our cohort. Despite these strategies to improve adherence, we still have a default rate around 20%. Rawson et al.^15^ previously presented their analysis of HIV patients treated for TB with RMP or RFB who also received ART, and found a higher default rate in the RFB group, perhaps because they started with RFB in the intensive phase deconstructing TB therapy or did not use RMP with lopinavir-ritonavir. Singh et al.^6^ found a lower though not significant difference in default rate when comparing HIV patients who used RFB, RMP or switched from RMP to RFB. In the study, patients used RFB, primarily with a PI-based regimen, and the standard dose was 150 mg three times a week, not 150 mg every day as is recommended in Brazil.^8^ ^,^ ^9^

In our study, the median initial CD4 cell counts were not significantly different between the groups, which probably justify the similar incidence of IRIS among patients who used RMP and RFB. Rawson et al.^15^ found a different result, with more cases of IRIS in the RMP group, possibly because these patients were severely immunosuppressed, though more susceptible to have IRIS.^16^ ^,^ ^17^ In Brazil, we have a low incidence of IRIS^18^ even starting ART early (data not published).

We had a low and identical percentage of TB treatment interruptions due to adverse reactions in both groups, unlike Rawson et al, who showed treatment interruptions were slightly more common in patients who used RFB in the British cohort.^15^ The authors of a Cochrane review did not find any significant differences in tolerability between the two drugs in five randomized controlled trials of predominantly HIV negative patients.^19^ Also, there were no significant differences in the overall number of patients who had severe adverse reactions according to type of rifamycin used in the study conducted by Singh et al.^6^ Chien et al.^20^ showed that RFB was well tolerated in most of non-HIV-infected patients who had previously experienced RMP-related adverse reactions that resulted in RMP interruption. In this group RFB-related hepatitis occurred in just 5% of patients; however, neutropenia was more frequent with RFB if compared to RMP.

Although patients who used RMP were more undernourished and presented more frequently with disseminated TB, they achieved better results of CD4 cell count and HIV viral load by the end of TB treatment compared to those who used RFB in our cohort. Most of patients who used RMP were ART-naïve, therefore had effective NNRTI regimen options. Patients who used RFB were ART-experienced and when the regimen already being used was not effective, there were few options to overcome HIV resistance, which made it difficult to treat. In the study of Rawson et al.,^15^ there were similar final CD4 cell counts and HIV viral load in RFB and RMP groups, perhaps because patients were better educated, and better comprehended the regimens prescribed, even in the RFB group.

When we compared patients who used RFB to the ART-experienced patients in the RMP group, we also found better outcomes of CD4 cell count and HIV viral load in the RMP group. Most ART-experienced patients in the RMP group were first-time ART users or never used NNRTI, and had the possibility to use this class of antiretroviral drug during TB treatment. However, most patients in the RFB group had already used several ART regimens and achieving a satisfactory immunological and virological response proved difficult.

TB was cured in a similar proportion of patients in both groups, possibly because treatment adherence and interruptions due to adverse reactions did not differ significantly.

Mortality was low and similar in both groups. This was also observed in other studies,^6^ ^,^ ^15^ that corroborate the importance of rifamycin in TB treatment and concomitant ART. We have already described that patients in this prospective cohort have higher mortality rates when rifamycins are not part of the TB treatment regime.^13^

This study has limitations. We compared a group of patients who used RMP including ART-naïve and ART-experienced patients, to a group of only ART-experienced patients who used RFB. In addition, the RFB group had fewer patients than the RMP group, what may have compromised the comparison of the groups. Since this was a cohort study, ART was prescribed to naïve patients, and modified in ART-experienced patients, based on the doctor decision and not on a date stipulated by a study protocol.

In conclusion, we found similar outcomes in HIV patients who used RMP or RFB as part of their TB treatment regimen, except for a worst immune and virological response in the RFB group, confirming the need for more effective treatment regimens for ART-experienced HIV-TB patients. The development of better strategies to treat TB and HIV in ART-experienced patients is urgent, as drug-drug interactions and therapy adherence are still a major concern and a limitation to both treatments.
